# Experimental Evaluation of Seed Limitation in Alpine Snowbed Plants

**DOI:** 10.1371/journal.pone.0021537

**Published:** 2011-06-29

**Authors:** Stefan Dullinger, Karl Hülber

**Affiliations:** 1 Department of Conservation Biology, Vegetation and Landscape Ecology, Faculty Centre of Biodiversity, University of Vienna, Vienna, Austria; 2 Vienna Institute for Nature Conservation and Analyses, Vienna, Austria; University Copenhagen, Denmark

## Abstract

**Background:**

The distribution and abundance of plants is controlled by the availability of seeds and of sites suitable for establishment. The relative importance of these two constraints is still contentious and possibly varies among species and ecosystems. In alpine landscapes, the role of seed limitation has traditionally been neglected, and the role of abiotic gradients emphasized.

**Methodology/Principal Findings:**

We evaluated the importance of seed limitation for the incidence of four alpine snowbed species (*Achillea atrata* L., *Achillea clusiana* Tausch, *Arabis caerulea* L., *Gnaphalium hoppeanum* W. D. J. Koch) in local plant communities by comparing seedling emergence, seedling, juvenile and adult survival, juvenile and adult growth, flowering frequency as well as population growth rates λ of experimental plants transplanted into snowbed patches which were either occupied or unoccupied by the focal species. In addition, we accounted for possible effects of competition or facilitation on these rates by including a measure of neighbourhood biomass into the analysis. We found that only *A. caerulea* had significantly lower seedling and adult survival as well as a lower population growth rate in unoccupied sites whereas the vital rates of the other three species did not differ among occupied and unoccupied sites. By contrast, all species were sensitive to competitive effects of the surrounding vegetation in terms of at least one of the studied rates.

**Conclusions/Significance:**

We conclude that seed and site limitation jointly determine the species composition of these snowbed plant communities and that constraining site factors include both abiotic conditions and biotic interactions. The traditional focus on abiotic gradients for explaining alpine plant distribution hence appears lopsided. The influence of seed limitation on the current distribution of these plants casts doubt on their ability to readily track shifting habitats under climate change unless seed production is considerably enhanced under a warmer climate.

## Introduction

The relative importance of abiotic constraints and seed limitation for the distribution and abundance of plants has received considerable interest during the last decade [1], [2], [3], [4]. In a recent meta-analysis of seed addition experiments, Clark *et al.* [2] demonstrated that seed limitation is a common phenomenon in plant populations when evaluated in terms of seedling numbers. These authors also found first-year mortality to be extremely high, however. As a corollary, they concluded that site factors, which control the transition from seedlings to subsequent life history stages, primarily restrict plant distribution patterns (establishment or site limitation). Nevertheless, definite conclusions about the roles of seed availability versus site suitability in limiting plant distribution requires following the fate of emerged seedlings until the reproductive stage [5], or to consider the relevant life history stages simultaneously. Such studies are few so far, mainly concern species with an annual life cycle and have delivered ambiguous results ([3], [4], [6]; but see [7], [8], for long-term assessments of perennial plants).

In high mountain landscapes the spatial distribution of plants has traditionally been explained by abiotic site conditions [9], [10] because environmental gradients are pronounced and often accompanied by abrupt shifts in species composition. On the other hand, seed limitation has been hypothesized to be particularly important in low-productive environments [11], [12] even if recruitment from seeds is not necessarily low in these systems [13](Zukrigl, 1999 #1463). Indeed, seed addition experiments in alpine habitats have demonstrated frequent seedling emergence at sites where adult con-specifics are not currently present. Although these studies only focus on early life history stages, like most comparable work in other environments, they nevertheless indicate that seed limitation is potentially important for the distribution and abundance of alpine plants.

Within the northern Calcareous Alps of Austria, snowbeds form particularly well defined habitat patches which cover only a small part of the landscape and harbour a specialized flora [14]. The availability of suitable sites is hence obviously crucial for the distribution of these snowbed specialists at the landscape scale. Nevertheless, in a recent analysis of patch occupancy patterns [15] we have demonstrated that rather than abiotic suitability patch size and connectivity explain their incidence and abundance in individual snowbeds. This result suggested that seed availability plays an important additional role for the incidence of snowbed plants in local communities. It has been argued, however, that assessing site suitability from abiotic conditions is error-prone because important factors might easily be missed. Experimental approaches are hence generally preferable [16]. Here, we thus evaluate the role of seed limitation for the incidence of snowbed species by comparing the performance of experimental plants in occupied, and hence obviously suitable, and unoccupied, and hence possibly unsuitable sites. Our test is based on the rationale that if site conditions primarily limit species occurrence plants should thrive significantly better in occupied than in unoccupied sites. On the other hand, independence of plant performance from site occupancy would provide a conservative indication of seed limitation (cf. [17]). To overcome the limitations of traditional seed addition studies we combined seed sowing with experimental transplantations of adults and juveniles and monitored seedling emergence, seedling, juvenile and adult survival, juvenile and adult growth, as well as flowering frequency over up to three vegetation periods.

Germination, growth and survival of species are not only determined by abiotic site conditions, however. Both negative and positive interactions with neighbors may additionally affect plant performance in alpine communities (e.g. [18], [19], [20]) with the relative importance of competition usually decreasing, and the importance of facilitation increasing with environmental severity [21], [22], [23]. Compared to other alpine habitats, snowbeds offer relatively benign site conditions because the long-lasting snow cover protects plants against climatic extremes [10], [24]. Consequently, negative plant-plant interactions have been shown to predominate in snowbeds [18] and snowbed specialists are sensitive to competition in terms of germination, growth and survival rates [25]. In our experiment, we hence additionally accounted for the possible effects of biotic interactions on these performance measures by integrating an indicator of neighborhood biomass into the analyses.

## Materials and Methods

### Study system

The study area is situated in the lower alpine zone (1850 to 1950 m.a.s.l.) of four neighboring mountain ranges (Mt. Schneeberg, Mt. Rax, Mt. Schneealpe, and Mt. Hochschwab, 15° to 16° E, and 47° 30′ to 47° 50′ N) of the northeastern Calcareous Alps of Austria. All four mountain ranges are part of the drinking water catchments of Vienna. Permission to conduct field work in the area was granted by the Vienna Water Management Department. Climatic conditions are temperate humid with a mean annual temperature of about 0–2°C and an annual precipitation between 1500 and 2500 mm in 2000 m.a.s.l. The topography of all four mountains is characterized by displaced plateaus at different altitudes. The upper subalpine zone is mainly covered by a krummholz belt of prostrate pine (*Pinus mugo* Turra) up to about 1850 m.a.s.l. Above the krummholz line, alpine grasslands and rock faces predominate. Within the grassland matrix, snowbed habitat patches occur on sites with a particularly long-lasting snow cover (∼8 to 10 months on average) such as, for example, small dolines, trenches or troughs. These snowbed patches are characterized by a sparse vegetation cover (31% on average across all patches surveyed, see below), and a high proportion of coarse scree material and rocks (49%). Organic and mineral soil horizons are shallow, if present. The vascular plant flora of such habitats is quite distinctive with small, rosette forming perennials representing the dominant life form [14].

From this vascular snowbed flora, we selected those four species that had highest germination rates in a preliminary growth chamber experiment - *Achillea atrata* L. (Asteraceae), *Arabis caerulea* L. (Brassicaceae), *Achillea clusiana* Tausch (Asteraceae) and *Gnaphalium hoppeanum* W. D. J. Koch (Asteraceae) - in order to be able to produce a sufficient number of juvenile plants for transplantation experiments (see below). All four species are insect-pollinated, clonally growing and rosette forming perennial herbs with a subalpine to subnival distribution and widespread on calcareous bedrock throughout the Alps, except for *A. clusiana*, which is endemic to the most northeastern Calcareous Alps.

### Experimental design

On each of the four mountains an area of 1.2×1.2 km was selected in the lab based on a digital elevation model and digital vegetation maps [26], [27], [28], [29]. Criteria for selection were among-area similarity in terms of altitude, location on a plateau, and the presence of a sufficient number of snowbed habitats according to the maps.

For each of these four areas, a complete fine-scale survey of snowbed patches was conducted in summer 2005. All 214 snowbeds identified were mapped by compassing their borders with a hand-held GPS and the whole area of each snowbed patch (average: ∼580 m^2^, min: 4 m^2^, max: 11000 m^2^) was searched for the presence of the four study species. From the total of 214 snowbeds, 55 (15 on Mts. Schneeberg, Rax, and Hochschwab; ten at Mt. Schneealpe) were picked for experiments. The selection aimed at simultaneously reducing both the proportion of snowbeds patches where the more frequent among the four study species were present and where the rarer species were absent, in order to increase chances of balanced occupancy rates of all species in the sample.

Within each of these 55 patches, we localized a plot of 3.0×2.5 m. We selected plot positions such that vegetation cover and substrate conditions were representative for those prevailing within the patch (but avoided solid rocks and coarse scree material which are hardly colonizable by our study species). Selection was done from a distant point at the patch margin, which allowed an overview of the whole patch but not of the distribution of individual species. After localizing the plots, the occurrence of the four species within a 5×5 m area around the plot center was recorded (*A. clusiana*: 72%, *G. hoppeanum*: 47%, *A. atrata* and *A. caerulea*: 20%). Plot corners were permanently marked, and the plot area was subdivided by a removable grid of 25×25 cm cell size. At the end of the experiment, in late summer 2008, vegetation height and cover was measured at five randomly chosen cells to get an indicator of neighborhood biomass at the individual plots.

In autumn 2005, three adult plants per study species, taken from snowbeds outside our four 1.2×1.2 km areas, were transplanted into three randomly selected individual cells of each experimental plot. Each transplanted adult was permanently marked with a colored thread. In addition, seeds of the study species were collected from different populations within the four mountain ranges both in autumn 2005 and 2006 and stored during winter under cold and dry conditions. In early April 2006 and 2007, a part of the seeds were germinated on wet filter paper in a climate chamber (15 h photoperiod, 90% humidity and a 23/15°C day–night cycle). The emerged seedlings were subsequently planted into small pots (diameter c. 2.5 cm, filled up to 2–3 cm with a substrate mixed of 1 part calcareous sand, 1 part compost and 2 parts mineral soil) and cultivated in the University of Vienna's common garden. Finally, the juveniles were transplanted into the experimental plots (together with the pot substrate) in early August 2006 and late July 2007, respectively. At the time of transplantation the above-ground biomass of plants was approximately equal to the one of juveniles during their third year under field conditions.

Our original design involved the sowing of 3×40 seeds and the transplantation of 3×3 juveniles of each study species into separate randomized cells of each experimental plot in both early summer 2006 and 2007. However, due to low seed set of *G. hoppeanum* and *A. caerulea* in the exceptionally cool growth period 2005 the intended design could only fully be realized for *A. atrata* and *A. clusiana*. For *G. hoppeanum* and *A. caerulea*, seed sowing and juvenile transplantation had to be restricted to a subset of plots in 2006 (*G. hoppeanum*: 28 plots, with only 2×40 seeds sown to each plot) or in both years (*A. caerulea*: 30 seed sowing and 29 juvenile transplantation plots in 2006, 38 seed sowing and 40 juvenile transplantation plots in 2007). Seeds were sown by spreading them into the existing vegetation. If the vegetation cover was sparse, we smoothly pressed the seeds onto the soil surface to avoid them being blown or washed away too easily. Transplanted juveniles were also marked with colored threads. Additionally, to estimate background seedling emergence three untreated control cells were randomly selected at each observation in each experimental plot.

Plots were re-visited five times in total (summer and autumn 2006, summer and autumn 2007, late summer 2008). At each visit, survival of transplanted juveniles and adults, as well as the emergence of seedlings from sown seeds and control cells were assessed. Emerged seedlings were not tagged individually to avoid damaging the tiny plants. Mapping of the seedlings was also impractical, because substrate movement on many plots caused seedling positions to change between re-visitations.

In addition, the following variables were measured for each transplanted adult and juvenile individual at the time of transplantation (autumn 2005, summer 2006 or summer 2007), and subsequently re-recorded in autumn 2006, autumn 2007, and late summer 2008: number of rosettes, number of leaves, length of the longest leave, the largest diameter of the major rosette and the diameter perpendicular on the largest one. Moreover, we assessed the presence/absence of reproductive shoots (inflorescence or infructescence). At the end of the experiment, the above-ground biomass of all alive transplants was harvested, oven dried at 60°C for two weeks, and their dry weight measured to the next mg. The relationship between the measured size variables and dry weight was estimated by means of least-squares regression using the variable values, and their possible two-way interactions, at the time of harvest in a forward selection procedure. Fitted regression models (*A. atrata*: F_6,603_ = 377.9, R^2^ = 0.79; *A. clusiana*: F_7,646_ = 326.5, R^2^ = 0.78; *A. caerulea*: F_8,225_ = 70.7, R^2^ = 0.72; *G. hoppeanum*: F_15,488_ = 121.8, R^2^ = 0.79, *p*<0.0001 in all cases) were then applied to predict the dry weight of all individuals at the time of transplantation and at each subsequent measurement date.

### Data analysis

Seven vital rates were derived from the measured data: seedling emergence, survival of emerged seedlings, transplanted juveniles and adults, growth of juveniles and adults, and sexual reproduction (combined for adults and juveniles). Seedling emergence was calculated in a conservative way: to avoid re-counting the same individuals, the number of seedlings newly emerged between two counts was defined as 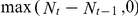
where N_t_ and N_t-1_ are the numbers of seedlings at the current and the preceding observation dates, respectively. Survival and reproduction were coded as binary attributes of each experimental plant. For the untagged seedlings, however, survival could not be monitored individually. To be consistent with the way we computed emergence, we assumed maximum seedling survival, i.e. the same number of seedlings in consecutive observations was interpreted as survival of all seedlings (no turn-over of individual seedlings). Growth was represented as the change in calculated dry weights between transplantation and each measurement date, respectively. To achieve symmetry around zero, this change was computed as the natural logarithm of the ratio between the weight at the respective observation date and the weight at transplantation. Thus, a value of zero reflects no change in biomass between two observations.

The seven rates were related to two independent variables, namely the occupancy of the plot by the respective species and the above-ground biomass of the surrounding vegetation estimated for this plot. Biomass was computed as the square root of the product of the mean vegetation height and the mean vegetation cover, averaged over the five measurements taken on each plot. Prior to running the statistical models, this measure was log-transformed in addition to achieve a near-normal distribution.

All our observations were grouped, i.e. several measurements were made on the same individuals (or in the same cells in case of seedling emergence and seedling survival) at consecutive observation dates. Individuals and cells, respectively, were moreover spatially blocked within mountain ranges and experimental plots. To account for this inter-dependence, we used mixed effects models for analysis and estimated a random intercept for every observation date and for each plot nested within the respective mountain range (for seedling emergence, seedling and adult survival, adult growth and reproduction) or for each cell nested in plots and mountains (for juvenile growth and survival). The random effect for cell was discarded, however, if model comparison (based on the Akaike Information Criterion (AIC)) did not indicate an improvement of the model (ΔAIC <2).

The type of mixed effects models used depended on the respective rate: We applied linear mixed effects models (LMMs), assuming a normal error distribution, for adult and juvenile growth, and generalized linear mixed effects models (GLMMs), assuming a binomial error distribution, in all other cases (i.e. the modeled responses were the probability of an individual to survive or to reproduce between two monitoring cycles, respectively the probability of a sown seed to germinate).

All LMMs and GLMMs were run both for all species in combination and for each species separately. In the first case, a separate random intercept for species identity was additionally included into the models. We also made trial analyses with random effects of plot occupancy and biomass for each species but in no case did AICs indicate that models were improved by these additional effects and we hence stayed with random intercept models.

### Matrix population models

To additionally derive an integrative measure of plant performance from the seven individual rates monitored, we computed the population growth rate λ from stage-classified transition matrices [30]. For each species, two 4×4 transition matrices were built comprising the pooled data of all occupied and unoccupied plots, respectively, from the two projection intervals 2006–2007 and 2007–2008 (cf. [31]). Separate matrices for each experimental plot could not be analyzed because data entries were too few in most cases.

A combination of size (number of leaves/rosettes and biomass) and reproductive criteria was used to distinguish four stage classes. We regarded individuals with only one rosette and not more than two leaves, i.e. the minimum size of a seedling at the end of the first growing season, as seedlings. The threshold-size separating juveniles and non-reproductive adults was defined as half the range of the biomass of the median 98% of transplanted individuals, i.e. one percent of the individuals of a species with the highest and lowest biomass were not accounted for to reduce the impact of outliers. Reproductive plants were classified as fertile (f) regardless of their size.

Because seedlings germinated from seeds artificially introduced into our experimental populations, and not from seeds produced by the experimental plants themselves, we did not relate fecundity to the number of fertile individuals in our experimental populations. Instead, we assumed that the number of seeds sown into one cell (40) is equivalent to the local seed shadow of one “standard” fertile individual and hence calculated fecundity, i.e. the adult-to-seedling transition rate, as the number of seedlings at the end of the projection interval divided by the number of sown-in cells.

Following Caswell [32], deterministic population growth rates λ (i.e. the dominant eigenvalue of the transition matrix) were then calculated and the λ values of occupied and unoccupied values were compared by means of non-parametric bootstrapping (1000 re-samples) of transition matrices (cf. [32], [33]). Differences in λ were considered significant if zero was not included within the 95% confidence interval of the bootstrap distribution.

Moreover, we constructed an elasticity matrix based on a transition matrix combining occupied and unoccupied plots following Caswell [32]. Each entry in the elasticity matrix measures the effect of changes in the corresponding element of the transition matrix on λ. Elasticity values were summed up as survival with no change of stage class (stasis), positive growth (progression to higher stage classes), negative growth (retrogression to lower classes), and fecundity to estimate the contributions of each of these processes to λ [34].

All statistical analyses were performed in R 2.8.0 [35] using the contributed packages lme4 [36] and boot [37].

## Results

### Individual rates in occupied and unoccupied cells

**Figure 1:**
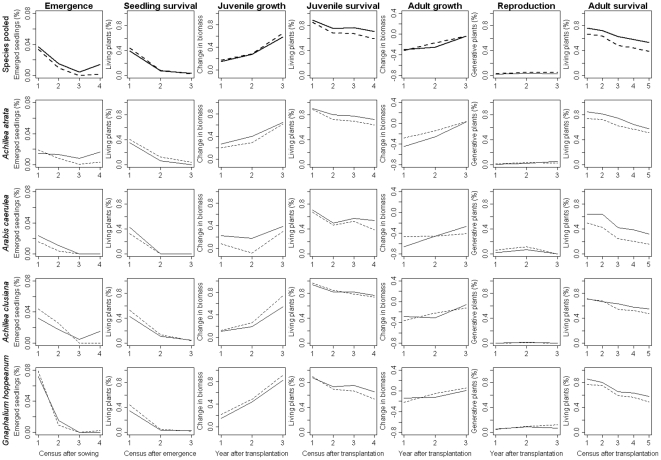
Vital rates of four snowbed plant species in the northeastern Calcareous Alps of Austria in plots occupied (solid lines) or unoccupied (dashed lines) by natural populations of the respective species. Growth was computed as the natural logarithm of the ratio between the weight at the respective observation date and the weight at the date of transplantation. For details see text.

Analyzing the data across all four species indicated that seedling emergence and adult survival rates of the experimental plants were higher in occupied than in unoccupied sites, whereas all other rates were independent of plot occupancy (Table 1). We note, however, that generative reproduction was very low for all species (Figure 1) and the power of the statistical test was hence limited for this performance measure.

**Table 1:** Fixed effect coefficients (FE, together with their standard errors, SE) of linear (juvenile and adult growth) or generalized linear (all other rates) mixed effects models relating the performance of experimental plants of four snowbed species of the northeastern Calcareous Alps (Austria) to their occurrence at experimental plots (occupancy) and to the biomass of the surrounding vegetation.

		*Achillea atrata*				*Achillea clusiana*				*Arabis caerulea*				*Gnaphalium hoppeanum*				*All Species*			
		FE	SE	*t*	*p*	FE	SE	*t*	*p*	FE	SE	*t*	*p*	FE	SE	*t*	*p*	FE	SE	*t*	*p*
Emergence	Occupancy	0,06	0,22	0,28	0,39	−0,20	0,25	−0,78	0,78	0,20	0,31	0,64	0,26	0,06	0,22	0,26	0,40	0,13	0,06	2,10	**0,02**
	Biomass	−0,38	0,15	−2,43	**0,01**	−0,39	0,20	−1,99	**0,02**	−0,43	0,22	−1,97	**0,03**	0,19	0,18	1,05	0,85	−0,25	0,14	−1,77	**0,04**
Seedling Survival	Occupancy	0,04	0,36	0,12	0,45	−0,48	0,41	−1,18	0,88	1,83	0,82	2,23	**0,01**	−0,06	0,38	−0,16	0,56	−0,03	0,11	−0,29	0,61
	Biomass	0,00	0,03	−0,12	0,45	0,00	0,04	0,07	0,53	0,05	0,07	0,76	0,78	−0,04	0,04	−1,11	0,13	0,18	0,70	0,25	0,60
Juvenile Growth	Occupancy	0,05	0,05	1,01	0,16	−0,05	0,05	−1,12	0,87	0,10	0,10	1,03	0,15	−0,01	0,06	−0,20	0,58	0,00	0,02	−0,16	0,57
	Biomass	−0,13	0,03	−3,94	**0,00**	−0,05	0,03	−1,61	0,05	−0,20	0,07	−2,86	**0,00**	0,02	0,05	0,50	0,69	−0,09	0,03	−3,41	**0,00**
Juvenile Survival	Occupancy	0,34	0,39	0,86	0,20	−0,14	0,34	−0,40	0,66	0,14	0,37	0,38	0,35	0,27	0,39	0,69	0,25	0,17	0,12	1,42	0,08
	Biomass	−0,32	0,25	−1,28	0,10	−0,05	0,24	−0,22	0,42	−0,37	0,27	−1,40	0,08	0,00	0,31	−0,01	0,50	−0,12	0,18	−0,70	0,23
Adult Growth	Occupancy	−0,19	0,09	−2,07	0,98	−0,02	0,10	−0,16	0,56	−0,08	0,12	−0,61	0,73	0,04	0,07	0,58	0,28	0,01	0,03	0,18	0,43
	Biomass	−0,02	0,01	−2,14	**0,02**	0,01	0,01	0,83	0,80	−0,03	0,01	−2,78	**0,00**	0,00	0,01	−0,12	0,45	−0,06	0,04	−1,49	0,07
Adult Survival	Occupancy	0,43	0,39	1,09	0,14	0,49	0,36	1,34	0,09	0,62	0,37	1,68	**0,04**	0,36	0,30	1,20	0,12	0,47	0,16	2,98	**0,00**
	Biomass	−0,38	0,26	−1,44	0,08	−0,19	0,27	−0,68	0,25	−0,17	0,24	−0,71	0,24	−0,57	0,26	−2,16	**0,02**	−0,30	0,15	−2,03	**0,04**
Reproduction	Occupancy	0,43	0,59	0,73	0,23	−1,33	1,34	−0,99	0,84	0,67	0,53	1,26	0,11	−0,02	0,31	−0,08	0,53	0,12	0,23	0,50	0,31
	Biomass	0,24	0,33	0,73	0,77	−0,25	0,92	−0,27	0,39	−0,14	0,37	−0,37	0,36	0,24	0,27	0,91	0,82	0,08	0,22	0,34	0,63

*t* is FE/SE and *p*-values are for one sided Student's *t*-tests of the hypotheses that occupancy has a positive and that biomass a negative effect on the respective rates.

Focusing on individual species demonstrates that seedling emergence, juvenile and adult survival of three species (*A. atrata*, *A. caerulea*, *G. hoppeanum*) were indeed consistently higher in occupied plots (Figure 1). However, in only one case, namely for the survival of adult *A. caerulea*, these differences were statistically significant when the data were analyzed for each species separately (Table 1). Moreover, this species, *A. caerulea*, was also the only one with statistically significant lower seedling mortality at occupied sites. The generic trends detected when analyzing the species altogether hence arose from weak and mostly insignificant, but parallel responses of three out of four species. Had the experiment been conducted with just one species, effects of plot occupancy on plant performance would have only been detected with *A. caerulea*.

Background emergence was very scarce. In 660 control cells per species, we found 2, 4 and 42 germinated seeds of *G. hoppeanum*, *A. caerulea* and the two *Achillea* species (which are hard to distinguish as first-year seedlings). In comparison, seedling numbers in the sown-in cells were 476, 420, and 1494 (*A. atrata*: 726, *A. clusiana*: 768), respectively. The low level of natural background emergence is hence unlikely to have affected our results.

### Response of individual rates to neighbor biomass

Plot biomass affected seedling emergence and adult survival, as did plot occupancy, and, in addition, juvenile survival when data were analyzed across all species (Table 1). For all these rates, the relationship with plot biomass was negative, i.e. plant performance decreased when the surrounding vegetation was more vigorous. As compared to the differences among occupied and unoccupied sites, this competitive neighborhood effect was also detectable when analyzing the species separately: emergence of three (*A. atrata*, *A. clusiana* and *A. caerulea*), juvenile and adult growth of two (*A. atrata* and *A. caerulea*) and adult survival of one (*G. hoppeanum*) species, and hence at least one rate of any of the four species, were significantly reduced within denser vegetation canopies. By contrast, seedling and juvenile survival as well as generative reproduction of all four species were consistently insensitive to plot biomass.

### Matrix models

**Figure 2:**
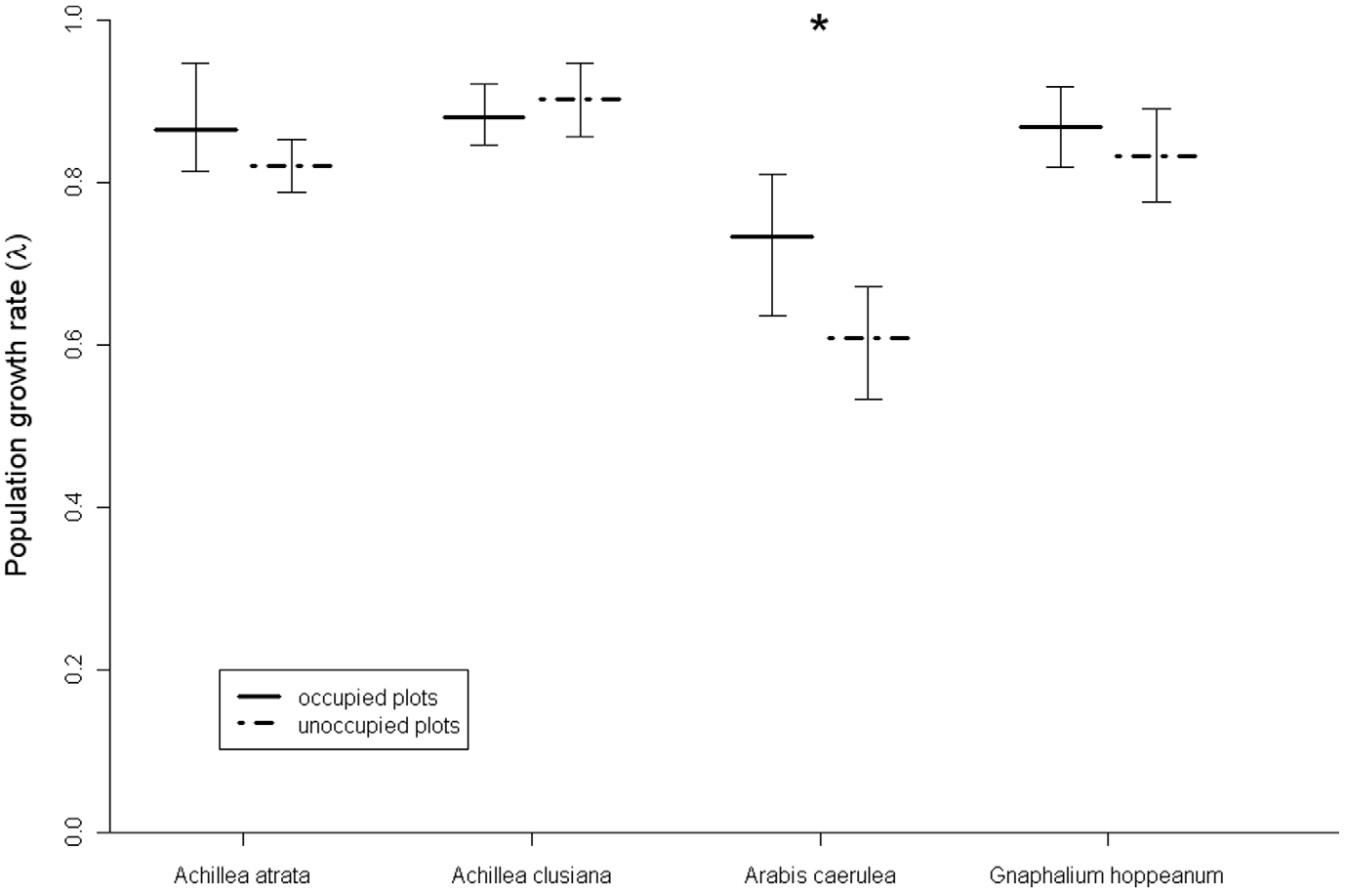
Growth rates (λ) of experimental populations of four snowbed plant species in the northeastern Calcareous Alps of Austria in plots occupied or unoccupied by natural populations of the respective species. The horizontal lines represent the means and the whiskers the 95% quantiles of λ values obtained by 1000 bootstrap resamples of the transition matrices of each species within either occupied or unoccupied plots. The difference in λ was considered significant (asterisk) if the 95% confidence interval of resample differences did not include zero.

Population growth rates λ of occupied and unoccupied plots differed significantly for one species only, *A. caerulea* (Figure 2). Absolute values of λ were below one throughout, but these absolute values are not interpretable in terms of natural dynamics due to the artificial character of the experimental populations.

The relative impacts of growth, survival and fecundity on λ were similar among study species. Survival was by far the most important demographic process for all species with elasticity values ranging from 0.61 to 0.69 (Table 2). Negative and positive growth had some additional effect whereas fecundity was unimportant.

**Table 2:** Relative contribution of demographic processes to population growth rates λ of experimental snowbed plant populations of the northeastern Calcareous Alps (Austria).

	negative growth	survival	positive growth	fecundity
*Achillea atrata*	0.163	0.642	0.189	0.006
*Achillea clusiana*	0.139	0.685	0.169	0.007
*Arabis caerulea*	0.181	0.614	0.198	0.006
*Gnaphalium hoppeanum*	0.128	0.619	0.228	0.025

Values represent elasticities, which were standardized following [42].

The sum of the elasticity values equals one for each species.

Overall, matrix models hence confirm the results obtained when analyzing individual rates – *A. caerulea* is the only species that has a clearly higher performance on occupied than on unoccupied plots; and it is mainly differential survival (of seedlings and adults) which drives the effect of plot occupancy on this species.

## Discussion

### Site vs. seed limitation

Taken together, our results show that one species, *A. caerulea*, thrives significantly better in occupied than in unoccupied sites, two (*A. atrata*, *G. hoppeanum*) show an at best weak response, and one species (*A. clusiana*) is largely insensitive to plot occupancy in terms of the measured rates and within the period of observation. The relative roles of site and seed limitation in driving the distribution of these four species hence obviously differ with seed limitation being evident in case of *A. clusiana* and limitation by site conditions clearly important for *A. caerulea*. This interpretation is consistent with a recent analysis of patch occupancy patterns of six snowbed plant species (the four included in this study and two additional ones) in the same study area: *A. clusiana* is the most common and *A. caerulea* the rarest species, most probably because *A. clusiana* is least, and *A. caerulea* most sensitive to variation in abiotic conditions among the individual patches [15]. Morevover, measurements of snow melt dates by means of temperature loggers buried directly at 30 of our 55 plots (loggers at the remaining plots unfortunately failed during the experiment) also suggested that the incidence of *A. clusiana* (and *G. hoppeanum*) at or around these plots is insensitive to melt-out dates, whereas *A. caerulea* (and *A. atrata*) prefer latest melting sites. As concluded by Moore & Elmendorf [3] for a very different ecosystem – annual plants in Californian grasslands – site and seed limited species can hence coexist in local communities. The relative importance of these two constraints on the incidence of a particular species depends on how wide its ecological tolerance, or niche, is in relation to the variation in environmental conditions realized in the system: the wider a specieś niche the lower the relative impact of site compared to seed limitation on its distribution [3].

Clark *et al.* [2] have partitioned seed limitation into source and dispersal limitation depending on whether the low production or the restricted spatial dissemination of propagules constrain their availability. The relative importance of both of these components of seed limitation is likely a matter of spatial scale with dispersal limitation more important at larger scales [17], [38]. In the studied system, both source and dispersal limitation probably contribute to plant distribution patterns. On the one hand, natural recruitment in the control plots was much lower than recruitment from sown seeds even in plots where adults of the study species were present in the natural canopy. Seed production rates seem hence insufficient to saturate sites even at the local scale. This finding is consistent with the low fecundity of our experimental plants even three years after transplantation (Figure 1) and with the scarcity of ripe seeds appropriate for experimentation on all four mountains in the cool and wet year of 2006. On the other hand, individual snowbeds are embedded into a grassland matrix and connectivity, and thus likely dispersal limitation, has been shown to co-determine the incidence of all four species at the landscape scale [15]. Source and dispersal limitation are, however, not independent of each other as source strength affects dispersal distances: the more seeds there are produced the more likely it is that even more distant and isolated sites are reached [39]. The relative roles of these two components of seed limitation are hence hardly quantifiable. Nevertheless, our data suggest that low rates of sexual reproduction at least contribute to restricted species distributions in these snowbed environments.

We note that the transplantation of juvenile plants together with their root balls eventually had some equalizing effect on the abiotic conditions experienced by these plants. However, we do not believe that such an equalizing effect was particularly influential in our experiment because, first, the amount of co-transplanted substrate was small; and, second, juvenile plants of the same four species which were pre-grown and transplanted in the same way as in this study proved highly sensitive to abiotic conditions in terms of germination, growth and survival along a snowmelt gradient from the centre to the margins of a large snowbed within the same study area [25].

### Importance of individual rates

In general, variation in the response of individual demographic rates to abiotic or biotic factors is not uncommon in plants [40], [41]. It is hence not surprising that conclusions about seed or site limitation can depend on the vital rates that are actually compared (see also [8]). Here, adult survival was the most sensitive indicator of site limitation, followed by seedling emergence and establishment. By contrast, had the experiment been focused on growth or reproduction rates, no differences between occupied and unoccupied plots for any of the species would have been detected. These results lend some support to the common focus on early life-history stages in studies of seed limitation [1], [2] but indicate that monitoring the survival of transplants would be a useful and feasible complement [7], [8]. This would be the more instructive as assessing seed limitation ultimately requires focusing on the establishment of viable local populations and survival is usually the vital rate most crucial for the long-term dynamics of long-lived plants [42], [43]. Indeed, the sensitivity of *A. caerulea* to plot occupancy in terms of λ was clearly driven by the differential mortality of both seedlings and adults in occupied and unoccupied plots.

### Neighborhood effects

Our results suggest that suitable, but unoccupied sites obviously exist in this snowbed system. However, this finding does not imply, of course, that all available sites are equally suitable to each species. In a recent study we have shown that even within a particular snowbed both abiotic conditions and neighborhood densities can strongly modify the performance of snowbed plants [25]. The results from the current experiment corroborate the sensitivity of snowbed species to competition. At least one vital rate of each of the four study species was significantly related to our indicator of aboveground biomass of the surrounding vegetation and all these responses were consistently negative. Competitive responses were detectable for seedling emergence (cf. [44]) and adult survival. Both of these rates were also responsive to plot occupancy. In addition, however, growth rates, which appeared completely unaffected by occupancy, were also sensitive to the neighbor biomass. The most likely explanation of this discrepancy is that competition affects the availability of resources that are essential for growth but do not differ among occupied and unoccupied plots. Radiation might be such a resource as light availability is effectively reduced by a denser vegetation canopy but hardly differs among individual snowbed sites. Belowground competition for nutrients, which likely also increases with a denser aboveground canopy, might play an additional role because plants on high mountain pioneer soils have been shown to be highly responsive to nutrient addition in terms of growth [45]. Whatever the reason, the detected effect of neighbor biomass on species performance corroborates that the site factors determining snowbed plant distribution comprise both abiotic conditions and the biotic environment [25], [46].

### Conclusions

Overall, our results suggest that seed and site limitation jointly determine the species composition of the studied snowbed plant communities and that constraining site factors include both abiotic conditions and biotic interactions. The traditional explanation of alpine plant distribution patterns by a particular, or exclusive, focus on abiotic niche constraints [9], [10] hence appears lopsided. Clearly, the importance of abiotic conditions will become the more detectable the more diverse the array of habitats under study – when comparing snowbeds with wind-swept ridges all species of the regional pool will primarily appear limited by the abiotic environment [10]. However, with a narrower focus on sites similar in environmental conditions, as in this study, the additional effect of seed limitation as well as of biotic interactions becomes obvious.

Seed limitation in alpine plant communities also has ramifications in applied contexts. For example, the possible fate of high mountain floras under predicted climate warming has repeatedly been assessed using so-called habitat distribution models (e.g. [47], [48], [49]). These habitat distribution models rely on an equilibrium between species and environment in the sense that they assume complete occupancy of suitable sites [50]. Their sensitivity to deviations from the equilibrium assumption has hardly been evaluated systematically but such deviations may potentially distort their projections of spatial habitat shifts under novel environmental conditions. Moreover, if alpine plants are seed limited even under current climatic conditions, their ability to readily track a rapidly shifting climate [51] seems questionable unless seed production is considerably enhanced under a warmer climate.

## References

[1] Turnbull LA, Crawley MJ, Rees M (2000). Are plant populations seed-limited? A review of seed sowing experiments.. Oikos.

[2] Clark CJ, Poulsen JR, Levey DJ, Osenberg CW (2007). Are plant populations seed limited? A critique and meta-analysis of seed addition experiments.. American Naturalist.

[3] Moore KA, Elmendorf SC (2006). Propagule vs. niche limitation: untangling the mechanisms behind plant species' distributions.. Ecology Letters.

[4] Moore KA (2009). Fluctuating patch boundaries in a native annual forb: the roles of niche and dispersal limitation.. Ecology.

[5] Myers JA, Harms KE (2009). Seed arrival, ecological filters, and plant species richness: a meta-analysis.. Ecology Letters.

[6] Emery N, Stanton ML, Rice KJ (2009). Factors driving distribution limits in annual plant community.. New Phytologist.

[7] Gustafsson C, Ehrlén J, Eriksson O (2002). Recruitment in Dentaria bulbifera: the role of dispersal, habitat quality and mollusc herbivory.. Journal of Vegetation Science.

[8] Ehrlén J, Münzbergova Z, Diekmann M, Eriksson O (2006). Long-term assessment of seed limitation in plants: results from an 11-year experiment.. Journal of Ecology.

[9] Billings WD, Mooney HA (1968). The ecology of arctic and alpine plants.. Biological Reviews.

[10] Ellenberg H, Leuschner C (2010). Vegetation Mitteleuropas mit den Alpen..

[11] Zobel M, Otsus M, Liira J, Moora M, Mols T (2000). Is small-scale species richness limited by seed availability or microsite availability.. Ecology.

[12] Foster BL (2001). Constraints on colonization and species richness along a grassland productivity gradient: the role of propagule availability.. Ecology Letters.

[13] Forbis TA (2003). Seedling demography in an alpine ecosystem.. American Journal of Botany.

[14] Englisch T, Valachovic M, Mucina L, Grabherr G, Ellmauer T, Grabherr G, Mucina L (1993). Thlaspietea rotundifolii.. Die Pflanzengesellschaften Österreichs Teil II.

[15] Dullinger S, Mang T, Dirnböck T, Ertl S, Gattringer A (2011). Patch configuration affects alpine plant distribution.. Ecography.

[16] Ouborg NJ, Eriksson O, Hanski I, Gaggiotti OE (2003). Toward a metapopulation concept for plants.. Ecology, Genetics, and Evolution of Metapopulations.

[17] Münzbergová Z (2004). Effect of spatial scale on factors limiting species distributions in dry grassland fragments.. Journal of Ecology.

[18] Choler P, Michalet R, Callaway RM (2001). Facilitation and competition on gradients in alpine plant communities.. Ecology.

[19] Klanderud K (2005). Climate change effects on species interactions in an alpine plant community.. Journal of Ecology.

[20] Klanderud K (2010). Species recruitment in alpine communities: the role of species interactions and productivity.. Journal of Ecology.

[21] Callaway RM, Brooker RW, Choler P, Kikvidze Z, Lortie CJ (2002). Positive interactions among alpine plants increase with stress.. Nature.

[22] Kikvidze Z, Pugnaire FI, Brooker RW, Choler P, Lortie CJ (2005). Linking patterns and processes in alpine plant communities: a global study.. Ecology.

[23] Dullinger S, Kleinbauer I, Pauli H, Gottfried M, Brooker R (2007). Weak and variable relationships between environmental severity and small-scale co-occurrence in alpine plant communities.. Journal of Ecology.

[24] Körner C (2003). Alpine plant life: Functional plant ecology of high mountain ecosystems..

[25] Hülber K, Bardy K, Dullinger S (2011). Effects of snowmelt timing and competition on the performance of alpine snowbed plants.. Perspectives in Plant Ecology, Evolution and Systematics.

[26] Greimler J, Dirnböck T (1996). Die subalpine und alpine Vegetation des Schneebergs, Niederösterreich. Vegetationskarte im Maßstab 1:10000 und Beschreibung der Vegetation.. Linzer biologische Beiträge.

[27] Dirnböck T, Greimler J (1997). Subalpin-alpine Vegetationskartierung der Raxalpe, nordöstliche Kalkalpen, Vegetationskarte 1:12500.. Linzer biologische Beiträge.

[28] Dirnböck T, Dullinger S, Gottfried M, Grabherr G (1999). Die Vegetation des Hochschwab (Steiermark) - Alpine und Subalpine Stufe.. Mitteilungen des Naturwissenschaftlichen Vereins der Steiermark.

[29] Pearman PB, Randin CF, Broennimann O, Vittoz P, van der Knaap WO (2008). Prediction of plant species distributions across six millennia.. Ecology Letters.

[30] Lefkovitch LP (1965). The study of population growth in organisms grouped by stages.. Biometrics.

[31] Horvitz CC, Schemske DW (1995). Spatiotemporal variation in demographic transitions of a tropical understory herb: projection matrix analysis.. Ecological Monographs.

[32] Caswell H (2001). Matrix population models: construction, analysis, and interpretation..

[33] Efron B, Tibshirani RJ (1993). An introduction to the bootstrap..

[34] Silvertown J, Franco M, Pisanty I, Mendoza A (1993). Comparative plant demography - relative importance of life-cycle components to the finite rate of increase in woody and herbaceous perennials.. Journal of Ecology.

[35] R Development Core Team (2008). A language and environment for statistical computing.. R foundation for Statistical computing.

[36] Bates D, Maechler M, Dai B (2008). http://lme4r-forger-projectorg/.

[37] Canty A, Ripley B (2008). boot: Bootstrap R (S-Plus). Functions.. R package version 1.

[38] Münzbergová Z, Herben T (2005). Seed, dispersal, microsite, habitat and recruitment limitation: identification of terms and concepts in studies of limititations.. Oecologia.

[39] Greene DF, Calogeropoulos C, Bullock JM, Kenward RE (2001). Measuring and modelling seed dispersal of terrestrial plants.. Dispersal ecology.

[40] Grubb PJ (1977). The maintenance of species richness in plant communities: the importance of the regeneration niche.. Biological Reviews.

[41] Howard TG, Goldberg DE (2001). Competitive response hierarchies for germination, growth, and survival and their influence on abundance.. Ecology.

[42] Franco M, Silvertown J (2004). A comparative demography of plants based upon elasticities of vital rates.. Ecology.

[43] Forbis TA, Doak DF (2004). Seedling establishment and life history trade-offs in alpine plants.. American Journal of Botany.

[44] Eskelinen A, Virtanen R (2005). Local and regional processes in low-productive mountain plant communities: the roles of seed and microsite limitation in relation to grazing.. Oikos.

[45] Heer C, Körner C (2002). High elevation pioneer plants are sensitive to mineral nutrient addition.. Basic and Applied Ecology.

[46] Heegard E, Vandvik V (2004). Climate change affects the outcome of competitive interactions - an application of principal response curves.. Oecologia.

[47] Dirnböck T, Dullinger S, Grabherr G (2003). A regional impact assessment of climate and land use change on alpine vegetation.. Journal of Biogeography.

[48] Randin C, Engler R, Normand S, Zappa M, Zimmermann NE (2009). Climate change and plant distribution: local models predict high-elevation persistence.. Global Change Biology.

[49] Engler R, Randin C, Thuiller W, Dullinger S, Zimmermann A (2011). 21st century climate change threatens mountain flora unequally across Europe.. Global Change Biology.

[50] Guisan A, Thuiller W (2005). Predicting species distributions: offering more than simple habitat models.. Ecology Letters.

[51] Engler R, Randin C, Vittoz P, Czáka T, Beniston M (2009). Predicting future distributions of mountain plants under climate change: does dispersal capacity matter?. Ecography.

